# Time-of-Day-Dependent Physiological Responses to Meal and Exercise

**DOI:** 10.3389/fnut.2020.00018

**Published:** 2020-02-28

**Authors:** Shinya Aoyama, Shigenobu Shibata

**Affiliations:** ^1^Graduate School of Biomedical Science, Nagasaki University, Nagasaki, Japan; ^2^Graduate School of Advanced Science and Engineering, Waseda University, Tokyo, Japan

**Keywords:** circadian rhythm, chrono nutrition, chrono exercise, time-restricted feeding, meal pattern

## Abstract

The mammalian circadian clock drives the temporal coordination in cellular homeostasis and it leads the day-night fluctuation of physiological functions, such as sleep/wake cycle, hormonal secretion, and body temperature. The mammalian circadian clock system in the body is classified hierarchically into two classes, the central clock in the suprachiasmatic nucleus (SCN) of the hypothalamus and the peripheral clocks in peripheral tissues such as the intestine and liver, as well as other brain areas outside the SCN. The circadian rhythm of various tissue-specific functions is mainly controlled by each peripheral clock and partially by the central clock as well. The digestive, absorptive, and metabolic capacities of nutrients also show the day-night variations in several peripheral tissues such as small intestine and liver. It is therefore indicated that the bioavailability or metabolic capacity of nutrients depends on the time of day. In fact, the postprandial response of blood triacylglycerol to a specific diet and glucose tolerance exhibit clear time-of-day effects. Meal frequency and distribution within a day are highly related to metabolic functions, and optimal time-restricted feeding has the potential to prevent several metabolic dysfunctions. In this review, we summarize the time-of-day-dependent postprandial response of macronutrients to each meal and the involvement of circadian clock system in the time-of-day effect. Furthermore, the chronic beneficial and adverse effects of meal time and eating pattern on metabolism and its related diseases are discussed. Finally, we discuss the timing-dependent effects of exercise on the day-night variation of exercise performance and therapeutic potential of time-controlled-exercise for promoting general health.

## Introduction

Several human physiological functions such as sleep/wake cycle, blood pressure, hormone secretion, body temperature, and physical activity exhibit around 24 h cycles called circadian rhythm. The anticipated diurnal change of a physiological function is also observed prior to the diurnal changes in environmental conditions such as light/dark cycle and temperature changes due to the rotation of the earth. This anticipative adaptation is driven by a circadian clock system existing in several tissues. The mammalian circadian clock system has an established hierarchy to distinguish between a central clock in the suprachiasmatic nucleus (SCN) of the hypothalamus and peripheral clocks in peripheral tissues including liver, lung, kidney, skeletal muscle and adipose tissue, as well as brain areas outside the SCN (1). The photic signal transmitted from the retina to SCN entrains the central clock, or master pace maker, that provides temporal cues to circadian clocks in the whole body. The temporal information of the central clock is transmitted to the peripheral clocks via neural and endocrine pathways, such as the sympathetic nervous system and glucocorticoid signaling (2, 3). The peripheral clocks are entrained by not only a light-induced signaling from the SCN but also other stimuli such as feeding, exercise, and stress in a SCN-independent manner (4–7). Nutrients entrain peripheral clocks (e.g., liver) via the activation of transcriptional and translational regulation of molecular clocks (see below) [for review, see (7, 8)]. For example, the ingestion of carbohydrate increases the insulin secretion, following the activation of the transcription and translation of clock genes and proteins (especially *Period2*), via the activation of insulin signaling (9, 10). Likewise, exercise entrains the circadian clocks in the peripheral tissues such as muscle, liver and lung via the sympathetic nervous system and glucocorticoid signaling (11, 12). These effects of nutrient and exercise on circadian clock are observed not only in rodents, but also in humans (13, 14).

**Figure 1:**
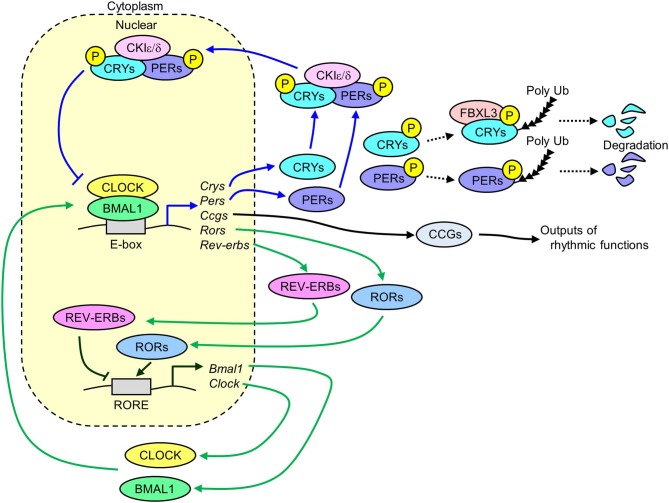
Transcriptional and translational negative feedback loop of core molecular clocks in mammals. The heterodimer of CLOCK and BMAL1 activates the transcription of *Crys, Pers, Rors, Rev-erbs*, and *Ccgs*. The translated and phosphorylated PERIODs and CRYs form a complex along with CKIε/δ and then this complex is translocated into the nucleus to inhibit its own transcription induced by the heterodimer of CLOCK and BMAL1 (blue lines). A part of phosphorylated CRYs and PERIODs is degraded via ubiquitin-proteasome pathways. The translated REV-ERBs and RORs inhibits and activates the transcription of *Bmal1* and *Clock* genes via binding to RORE, respectively (green lines). The rhythmic expression of *Ccgs* results in oscillation of several physiological functions (Crys, Cryptochrome1/2; Pers, Period1/2; Rors, Retinoid-related orphan receptors; Rev-erbs, reverse-Erb receptors; Ccgs, Clock-controlled genes; CKIε/δ, Casein kinase 1ε/δ; RORE, retinoic acid receptor response element).

The molecular mechanisms of circadian clock systems in mammals have been investigated since the discovery of *Clock* gene (*Circadian locomotor output cycles kaput*) in 1997 (15). Several core clock genes have been identified in mammals, including *Bmal1* (*Brain and muscle ARNT-like 1*), *Clock, Per1* (*Period1*), *Per2, Cry1* (*Cryptochrome1*), and *Cry2*. These genes interact with each other via transcriptional and translational negative feedback loops to exhibit a 24 h cycle (Figure 1). The heterodimer of CLOCK and BMAL1 works as transcriptional factors and has a basic helix-loop-helix PAS domain. The binding of this heterodimer to an E-box binding element in the promoter regions of *Pers* and *Crys* activates the transcription of these genes (16). The translated PER1/2 proteins are phosphorylated by CKIε/δ (Casein kinase Iε/δ) in the cytoplasm (17). The phosphorylated PER1/2 proteins are unstable and are degraded by the ubiquitination-proteasome pathway (18, 19). Similar degradation is seen in the CRY1/2 proteins due to ubiquitination systems via FBXL3 (F-box and leucine rich repeat protein 3) (20). The CRY1/2 and PER1/2 proteins in the cytoplasm promotes the formation of PERs/CRYs/CKIε/δ complex. This complex then transfers to the nucleus and suppresses the transcription induced by the heterodimer of CLOCK and BMAL1. The transcription of *Clock* and *Bmal1* is negatively and positively controlled by REV-ERBs (nuclear receptor subfamily 1, group D) and RORs (RAR-related orphan receptor), respectively via binding to a ROR-responsive element (21, 22). Similarly, *Pers* and *Crys*, the *Rev-erbs* and *Rors* genes are also the target of the BMAL1 and CLOCK complex (21, 22). The BMAL1/CLOCK complex temporally controls the transcription of other genes, which are called clock-controlled genes (CCGs), such as *Dbp* (*D-site of albumin promoter binding protein*) and *Ppar*α (*Peroxisome proliferator activated receptor* α) via binding to respective responsive element sequences (23–25). This negative feedback loop of clock genes exists in nearly all tissues in mammals.

Circadian transcriptomics revealed that the expression of rhythmic genes occurs in a tissue-specific manner (26, 27). Through the analysis of mice with tissue-specific clock gene mutations, the importance of peripheral clocks in tissue-specific functions is being revealed (28, 29). Especially, nutrient metabolism exhibits a clear day-night variation in tissues with high metabolic activity, such as liver, muscle and adipose tissue, where its diurnal change is directly regulated by an intrinsic clock (30–34). From these results, it is thought that one of the major roles of the peripheral clock is to prepare for the transition from the rest phase to the active phase, and responding to high energy demand (26, 30, 31, 35). Considering that the metabolic process of each nutrient is diurnally controlled, it is expected that the postprandial response of metabolic functions depends on the feeding time. Also, fuel selection in a skeletal muscle during exercise depends not only on the nutritional state, but also on the time of day (36, 37). Additionally, some of exercise-regulated factors such as AMPK (AMP-activated protein kinase) are temporally activated by circadian clocks (38). In this review, we discuss the time-dependent physiological response to nutrients and a role of circadian clock in these time-dependent effects. Finally, we also summarize the time-dependent effects of exercise on physiological functions and athletic performance.

## Time-of-Day-Dependent Postprandial Response of Macronutrients

Generally, we take meals three times a day. Although there are many reports focusing on the postprandial metabolic response to a single meal, it is rare that the research focuses on the comparison between metabolic responses to breakfast, lunch, and dinner. In this section, we review the time-of-day effects on the postprandial metabolic responses of macronutrients and the influence of circadian rhythm to these time-of-day effects.

### Lipid Metabolism

It is observed that the postprandial triacylglycerol (TG) response is dependent on the eating time. Sopowski et al. investigated the blood TG response to identical high-fat meal consumed during the daytime (13:30) and the night time (01:30) in healthy men and women. They reported higher and longer postprandial elevation of TG at the night time than that at daytime (39). The meal-time-specific postprandial response of blood TG is also different between breakfast and lunch, and the increasing of TG levels after lunch is ~2-fold less than that after breakfast in men (40). A weak postprandial response to lunch is also exhibited when breakfast had been skipped, suggesting that the endogenous circadian rhythm is involved in the differential effects observed after breakfast vs. lunch and dinner. In addition, addition of stable-isotope-labeled palmitic acid to the test meal was used to distinguish between the meal-derived TG and endogenous TG. The postprandial labeled-palmitic-acid level was not changed between breakfast and lunch, suggesting that the lower response of blood TG level after lunch involves fatty acids derived from endogenous sources but not the meal itself (40). Insulin suppresses the release of free fatty acids from adipose tissue (41). Considering that the change of insulin level also depends on the meal time, it is suggested that the lower elevation of TG after lunch could be dependent on insulin levels. The day-night variation of postprandial TG levels is reported in studies carried out on animal models, and the higher response of postprandial TG at the rest phase compared with the active phase is attributed to lower uptake of fatty acids into skeletal muscles and brown adipose tissues (42). This study reported that the postprandial day-night variations are not observed in SCN lesioned rats. Furthermore, lipid utilization is also directly regulated by the intrinsic muscle clock (43). Thus, it suggests that the circadian-clock-driven day-night variation of lipid uptake and utilization is related to the difference of postprandial TG response among meals. Recently, it was observed that the preventive effects of fish oil on hepatic steatosis and hyperlipidemia depend on feeding time in mice (44). In this study, Oishi et al. developed and used the two-meals-per-day feeding model. The blood levels of docosahexaenoic acid (DHA) and eicosapentaenoic acid (EPA) are higher in the mice fed with a fish oil during the time of activity onset compared with that during the onset of inactive phase, suggesting that feeding-time dependent therapeutic effects of fish oil rely on the temporal capacity of intestinal absorption of DHA and EPA.

### Glucose Metabolism

Like the effects observed on blood TG levels, the postprandial glucose levels exhibit a time-of-day-dependent response to meals. Glucose tolerance is higher in the morning than in the evening, in humans (37). It is known that the difference of glucose tolerance between the morning and evening is due to the temporal regulation of glucose utilization and pancreatic β cell function (see below). In fact, the dysregulation of glucose metabolism is observed in the whole-body or liver-, muscle-, or pancreatic βcell-specific clock gene mutant mice (30, 32, 33, 45, 46). In pancreatic β cells, a circadian clock controls the rhythmic transcription of insulin-secretion-related genes, and the decrease of a nutrient-induced insulin secretion is observed in the pancreatic β-cell-specific *Bmal1* knock out mice (45). Glucose uptake and utilization in peripheral tissues such as liver and skeletal muscle are differentially regulated (30, 32). For example, the murine muscle clock temporally regulates glucose uptake into skeletal muscle via the recruitment of GLUT4 (glucose transporter 4) to the plasma membrane and increased expression of glycolytic genes. Considering that this temporally regulated surge is observed prior to the active phase, it is thought that the muscle clock has a role in preparation for high energy demand at the beginning of active phase. In addition to animal studies, Morris et al. reported that the human intrinsic circadian system affects day-night variation of glucose tolerance and insulin-secretion by the use of circadian alignment and misalignment protocols (47). The magnitude of its effect is larger than effect of behavioral rhythm such as sleep/wake cycle and fasting/feeding cycle in humans. In addition, circadian misalignment between endogenous and behavioral rhythms also exacerbates glucose tolerance in shift workers (48), thus highlighting the importance of alignment between both rhythms for prevention of diabetes in shift-workers. Thus, the diurnal variation of glucose tolerance is regulated by both endogenous and behavioral rhythms, and the molecular clock in peripheral tissues drives endogenous rhythms such as glucose uptake and insulin secretion.

### Amino Acid Metabolism

There have been studies focused on the feeding-time-dependent postprandial response of amino acids and peptides and the diurnal absorptive capacity of these nutrients. In the small intestine of rodents, the absorption of some amino acids and peptides was activated in the early active phase rather than in the early rest phase (49). H^+^-coupled peptide transporter (PEPT1) is localized at the apical membrane of intestinal epithelial cells and has major role for di- or tri-peptide transportation in the small intestine. Pan et al. reported that the absorption of glycyl-sarcosine, which is one of the substrates of PEPT1, depends on the administration time in rodents (49). The blood glycyl-sarcosine level is higher after its administration in the early active phase than that in the early rest phase (49). In addition, the PEPT1 mRNA and protein levels in the rat duodenum and jejunum exhibit day-night variation and are elevated before active phase (49, 50). The pattern of day-night variations of PEPT1 level is associated with the diurnal pattern of glycyl-sarcosine uptake in the duodenum (49), suggesting that the diurnal variation of PEPT1 levels is involved in the time-dependent absorption of peptides in small intestines. Pan et al. also reported that another PEPT1's substrate, the antibiotic ceftibuten, is absorbed in a time-dependent manner in rodents (51). The time-dependent absorption and the diurnal rhythm of PEPT1 level are not observed under the fasting condition, suggesting that feeding cycle is important for the time-dependent effect. In fact, time-restricted-feeding led to a shift in the phase of diurnal PEPT1 level in the duodenum of rats (52). Albumin D site-binding protein (DBP) is one of the clock-controlled genes and its transcription is activated by the heterodimer of BMAL1 and CLOCK and suppressed by PERs and CRYs (53). In addition, DBP activates the transcription of several genes including *Pers* via DBP binding site and the expression of target genes shows the diurnal rhythmic pattern (53). *Pept1* has a DBP binding site in its promotor region. A luciferase assay designed using the promotor region of *Pept1* showed that DBP activates PEPT1 promotor activity (54). Okamura et al. reported that bile-acid-regulated PPARα activity leads to the diurnal expression of *Ppet1* in the intestinal cells of mice (55). The feeding-fasting cycle induced the day-night variation of cholic acid in the intestinal epithelial cells. Cholic acid decreases the *Pept1* levels before active phase, which corresponds to the peak time of *Pept1* expression, but not before rest phase, which is its trough time. The cholic-acid-dependent regulation of *Pept1* expression is suppressed by knockdown of PPARα. In addition, time-dependent absorption of carnosine, which is one of the substrates of PEPT1, is also not observed in PPARα-null mice. These reports suggest that absorption of peptides in small intestine exhibits diurnal variation via the DBP- and PPARα-mediated circadian control of PEPT1 expression. In recent years, time-dependent intestinal absorption of amino acids has been reported (56, 57). Jando et al. reported that isoleucine absorption is higher in the active phase than in the rest phase, although the protein levels of intestinal amino acid transporter B0AT1 in the rat intestine are not changed between the two time points (57). This study suggested that circadian expression and/or post-transcriptional modulation of other amino acids transporters is involved in the time-dependent intestinal isoleucine absorption. The branched-chain amino acids, such as leucine, valine, and isoleucine, are absorbed via LAT4 (SLC43A2), a basolateral neutral amino acid transporter (58). LAT4 phosphorylation at Ser274 is higher at the beginning of the rest phase than at the beginning of the active phase in mice (56). LAT4 shows high activity under dephosphorylated condition, suggesting that post-translational modulation such as phosphorylation could be involved in the time-dependent amino acid absorption. In studies involving human subjects, comparing the postprandial response between morning and evening using metabolomics, revealed that 16 amino acids such as arginine and leucine were detected at higher levels in blood in the morning than in the evening (59). These data suggest that the postprandial amino acids response in humans depends on the feeding time.

## Effects of Time-Restricted-Feeding

The feeding activity of a rodent is rhythmic and occurs mainly during the active phase, especially in the early active phase. The perturbations of feeding rhythm relate to the metabolic dysfunctions, leading to the onset of obesity, diabetes and lipidosis (60–63). Feeding a high-fat diet dampens the diurnal feeding/fasting cycle, resulting in more food intake during the inactive phase (64). The restriction of feeding time prevents the high-fat diet induced metabolic disorders, such as excessive body weight gain, glucose intolerance, hepatic steatosis, and inflammation (65, 66). Considering that the time-restricted feeding (TRF) also protects against the high-fat-diet-induced dampening of clock genes such as *Per2, Bmal1, Rev-erb*α, and *Cry1* in the liver (65), it is contemplated that the TRF prevents several metabolic dysfunctions via a rescue of rhythmic peripheral clock gene expression. However, the preventive effects of the TRF are also observed without changes of locomotor activity or calorie intake, in mice lacking a circadian clock, such as whole-body *Cry1/2* double knockout, liver-specific *Bmal1* or *Rev-erb*α*/*β knockout mice (67). Transcriptomic analysis of different mouse lines reveals that the transcripts to observed to be oscillating in the wild type mice under the TRF are mostly unaffected in the clock gene deficient mice under the TRF, thus suggesting that one of the main effects of TRF in clock gene deficient mice is maintaining basal level of gene expression rather than the temporal control of expression. In addition to gene expression profile, Chaix et al. discuss the possibility that the TRF may regulate a temporal post-translational modification because the TRF drives the oscillation of post-translational modification with greater amplitudes rather than those of transcripts and metabolites (68). Contrary to these studies, some reports showed that TRF had no effect on body weight loss in rodents (69–71). For example, TRF (12 h feeding window) during the light or dark phase did not change the body weight as compared to *ad libitum* feeding in rats (70, 71). Although the reason for these conflicting results is unclear, it is possible that the effects of TRF on body weight loss may depend on the periods of feeding window and diet composition in animal studies. In fact, the beneficial effects of TRF on body weight loss were especially observed in the case of high-fat diet feeding or under the shorter feeding windows (<8 h) (72). The preventive effect of TRF due to a reduction of meal frequency or a shortened feeding window is also observed in human studies (36, 73, 74). Sutton et al. reported the strict early-time-restricted feeding (6 h feeding window, with dinner time before 1500 h) for 5 weeks improves the insulin sensitivity and β cell function, blood pressure, and oxidative stress in prediabetic men (75).

**Figure 2:**
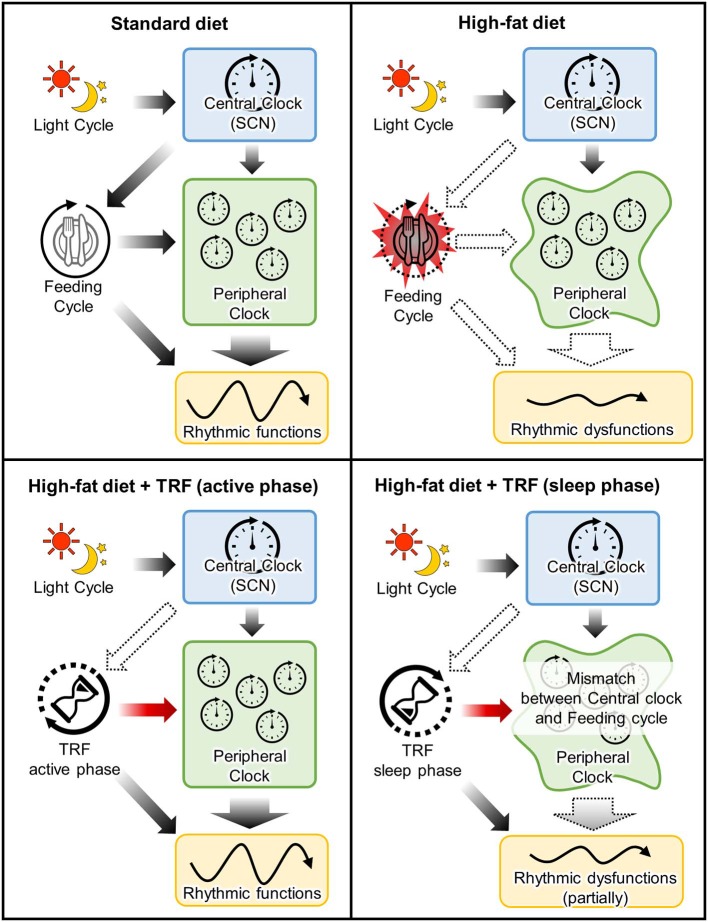
Mechanistic insight into the effects of time-restricted-feeding during active or sleep phase. Upper left panel: In standard-diet-fed mice, timing of the entrainment cues between feeding cycle and central clock is matched for peripheral clocks. Upper right panel: High-fat-diet not only induced metabolic dysfunction due to a high calorie but also arrhythmic feeding cycle. This dysregulation of feeding cycle attenuates the feeding-induced-entrainment of peripheral clocks. Lower left panel: TRF during active phase rescues the attenuation of peripheral clock functions due to perturbation of feeding cycle. Lower right panel: Although TRF during sleep phase also entrains peripheral clocks, timing of the entrainment cues between feeding cycle and central clock is mismatched. It is suggested that this mismatch partially attenuates the beneficial effects of TRF.

As mentioned before, food intake is one of the major non-photic entraining impulses in peripheral clocks in the peripheral tissue such as liver and adipose tissue, while it does not entrain a central clock in the SCN (7). Shift of calorie intake time to the sleep-phase induces desynchronization between peripheral and central clocks (76). In other words, the disturbance between fasting/feeding cycle and sleep/wake cycle leads to the disconnection between the central and peripheral clocks, resulting in induction of several metabolic dysfunctions (60, 61, 77–80) (Figure 2). For example, the larger food consumption at night or the delayed onset of feeding time due to breakfast skipping, is related to body weight gain and insulin sensitivity in humans (77, 78, 81–85). The adverse effects of rest-phase-feeding are observed in experimental animal models and it has been shown that the rest-phase-feeding-induced weight gain is induced without any change of locomotor activity and food intake (60). Additionally, some researchers have developed two- or three-meals-per-day-feeding models in rodents (meals in the early, middle, late active phase are defined as breakfast, lunch and dinner, respectively) to imitate general human meal pattern (86–88). The breakfast skipping rats had larger weight gain when compared with the dinner skipping rats (88). Larger weight gain is also observed in the delayed breakfast model without a change of total food intake (87). Also, Wu et al. showed that the beneficial effects of calorie restriction depends on which meal you restrict the calories from Wu et al. (88). Greater weight loss, circumference reduction, insulin sensitivity index, and triglyceride levels are observed in obese women who restricted calories from dinner compared to those who restricted it from breakfast (89). It suggests that consuming high calorie meal during the night induces the dysfunctions of lipid and glucose metabolisms even if the feeding window is shortened, like in time-restricted feeding.

In recent years, it is known that the feeding in the rest phase affects not only metabolic diseases but also other functions. Muscle mass is decreased by the rest phase feeding via the inactivation of IGF-1 signaling (90). The murine muscle growth and protein synthesis are down-regulated byTRF in the rest phase compared with the TRF in the active phase (91). In the murine skin, the rest-phase-TRF shifts the phase and reduces the amplitude of clock genes, leading to the dysregulation of the diurnal sensitivity of UVB-induced DNA damage and a key DNA-repair-related gene (92). On the other hand, the diurnal regulation of DNA synthesis is not affected by TRF. Thus, the rest-phase-TRF leads to the mismatch of temporal regulations between DNA synthesis and repair, resulting in the increased sensitivity to UVB-induced DNA damage. In summary, TRF during the optimal time, to avoid the sleep phase, could be effective for the maintenance of several biological functions, while TRF during the sleep phase might attenuate muscle and skin functions as compared to TRF during the active phase.

## Time-of-Day-Dependent Physiological Responses to Exercise

### Diurnal Variation of Physical Performance

Athletic performance such as muscle strength and endurance exhibits day-night variations (93–95). Generally, the human athletic performance is low in the morning and its peak time is late afternoon (93–95). Its diurnal change is closely related with the change of body temperature (96, 97). A hot environment blunts the day-night variation of muscle performance, such as muscle force, power, and contractility, thus it is thought that the body temperature partially contributes to the diurnal variation of physical performance (98). The circadian clock drives the oscillation of various physiological functions including body temperature (99). In addition to the body temperature, the diurnal pattern of human physical performance is also changed by chronotype, and its amplitude is greater in the evening type persons with a lower performance in the morning (100). The chronotype-specific day-night pattern is also observed in the swimming performance (101). Endurance exercise capacity is changed in mice with some clock gene deletions, such as *Rev-erb*α and *Cry1/2* (102, 103), suggesting the regulation of exercise performance by circadian molecular clock. In fact, Ezagouri et al. report that the diurnal variation of exercise capacity in mice relies on the clock proteins PER1/2 and they discover 5-aminoimidazole-4-carboxamide ribonucleotide (ZMP), which is AMPK activator, as a key factor to induce the time-specific effects of exercise on exercise capacity using both transcriptomic and metabolomic analyses (104).

**Figure 3:**
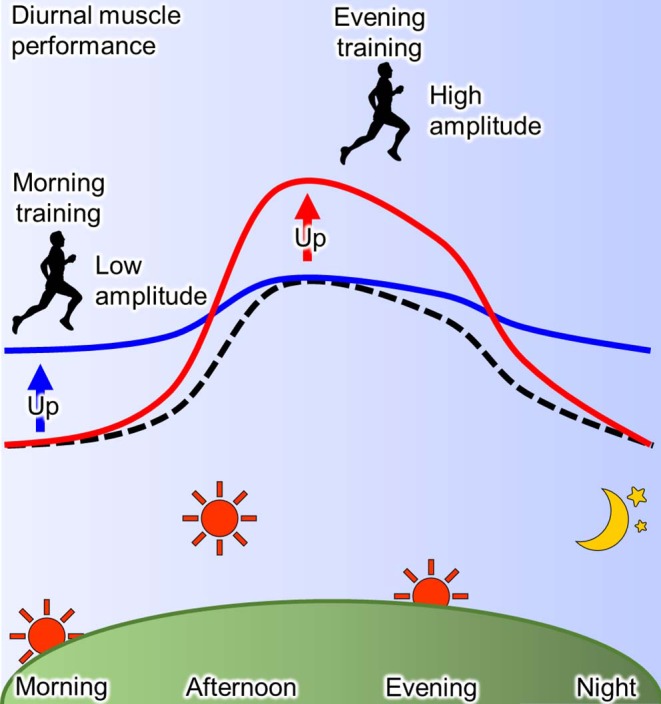
Scheme for effect of training-time on diurnal physical performance. The muscle performance exhibits diurnal variation with the peak at afternoon and the trough at morning and night (dotted line). This diurnal characteristic of muscle performance is changed by training and its effect depends on the time of day (93). Morning training increases the performance in morning resulting in the low amplitude (blue line), while evening training increases it in the evening resulting in the high amplitude (red line).

The training time within a day affects the day-night variation of human physical performance (93, 105). As mentioned before, human muscle power exhibits a diurnal variation, where it is lower in the morning than in the evening (93, 94). In a human study, this diurnal variation of the muscle power is blunted by 12 weeks of resistant training in the morning via an enhancement of muscle power (106). The reduced daily fluctuation of muscle power due to the exercise training is specific to morning training, while it is not observed in the evening training (93, 107). On the other hand, the exercise training in the evening induces the elevation of muscle performance in the afternoon, thus the magnitude of diurnal muscle performance change is increased in humans (107, 108). In summary, a high amplitude of muscle performance within a day is observed by training in the evening, while training in the morning decreases amplitude of daily muscle performance through the enhancement of performance in the morning (Figure 3). Similarly, in elite college basketball players, the performance in afternoon is lower during the morning training periods when compared with the afternoon training period, although the performance in the morning was not evaluated (109). These observations suggest that similar response to time-specific training is seen not only in common people, but also among elite athletes. In addition, it is possible that these changes of diurnal pattern are linked to competition ability. The times recorded for 200 m swimming trail in the morning were faster in the subjects who habitually train in the morning compared with those who train in the evening (101). From a practical point of view, Chtourou et al. recommend that the time-controlled training should be adjusted to same time of competition for exerting the best performance in the competition (93, 106). However, some reports show no effects of training time on the day-night variation of muscle performance (110, 111). It is a possibility that the different training conditions, such as duration and intensity, and the passive warm-up effect of the environment may have influenced the results.

### Blood Pressure and Blood Circulation

Blood pressure also exhibits a clear circadian rhythm with a lower blood pressure during the rest phase, increasing around the time of waking up, and the highest at active phase in human and rodents (112, 113). The time-dependent hypotensive effect of exercise was observed a long time ago in humans (114). Helen et al. reported the time of day effects on the post-exercise response of blood pressure in normotensive men (115). Cycling exercise at 60% VO_2max_ in the early morning (0400 h) induces the transient elevation of blood pressure while the exercise in the afternoon, evening, and night, do not change or transiently reduce blood pressure compared with each pre-exercise condition (115). Although it suggests that the morning exercise is not better for the reduction of blood pressure, Helen et al. do not evaluate the blood pressure under the sedentary conditions at each time point (115). Thus, the possibility remains that the reducing effect of morning exercise on blood pressure may be masked by the circadian rising of blood pressure in the morning, called “morning surge.” De Brito et al. evaluated the net change of post-exercise blood pressure, with the use of adjustment by the day-night variation of blood pressure, under the control sedentary condition in normotensive subjects (116). In the adjusted conditions, the post-exercise reduction of blood pressure is observed in both morning and evening, and its reduction is greater in morning than in the evening (116). In addition to blood pressure, the exercise-induced reduction of cardiac output and the weak response of exercise-induced increased heart rate are observed after morning exercise, while the sympathovagal balance and lower limb blood flow responses are increased after evening exercise (116). It suggests that the greater effect of morning exercise is due to cardiac and autonomic functions. Recently, it was reported that the responses of peripheral blood flow and vascular conductance after exercise are not changed between the morning and evening exercise in young subjects, suggesting that the time-of-day effects of exercise on blood pressure and vasodilation are likely reflecting central rather than peripheral regulation (117). Similarly, the higher response of blood pressure to cold exposure in hypertensive adults is reduced by the exercise in the morning but not in the evening (118), suggesting that the morning exercise promotes the reactivity of vasodilation.

The chronic anti-hypertensive effects of exercise training in the morning or evening on blood pressure were observed in anti-hypertensive-drug-treated men (119). Evening exercise training for 10 weeks (3 times a week) reduces systolic blood pressure and diastolic blood pressure during sleep, while their effects are not observed in morning trained hypertensive men. In contrast to the beneficial acute effects of morning exercise (116), the chronic effects of morning exercise are not observed. It is possible that the effect of anti-hypertensive drug masks the hypotensive effect of exercise in the morning, because all subjects took medication in the morning (119). Moreover, the net change of blood pressure is not evaluated under the adjusted condition described before, thus it is possible that some effects of morning exercise mask by the morning surge.

The timing-dependent hypotensive effect of exercise depends on the circadian characteristic of blood pressure. Park et al. investigated the hypotensive effect of exercise in the dipping or non-dipping hypertensive subjects (120). The dipping hypertensive subjects show a clear circadian rhythm of blood pressure, while the non-dipping subjects did not show it due to a less dropping in night-time blood pressure. The morning exercise reduces blood pressure with similar efficacy in the dipping and non-dipping hypertensive subjects. On the other hand, greater reduction of night-time blood pressure due to evening exercise was observed in non-dipping hypertensive subjects than in cases of dipping hypertensive subjects. Thus, it suggests that the timing of exercise is more important for controlling dipping hypertension rather than for non-dipping hypertension. Based on this research, it is expected that the evening exercise has beneficial effects in non-dipping hypertensive men. However, in a recent human study, exercise at the evening and night-time (from 1900 to 2200 h) delays the phase of melatonin metabolites (14), thus it is possible that evening exercise progress the circadian disturbance of blood pressure via a phase-delay of circadian rhythm. Further studies are required to evaluate the effect of exercise both in terms of hypotensive effect and circadian rhythm is required for the control of circadian blood pressure in hypertensive subjects.

### Muscle Size

The exercise training increases and/or maintains muscle size via controlling the balance of muscular protein turnover (121, 122). The combination of endurance and resistance training for 24 weeks induces muscle hypertrophy, and along with training in the evening leads to larger magnitude in muscle cross sectional area compared to the same training in the morning in men (111). Sedliak et al. reported similar effects of the time-of-day-specific resistance training on muscle mass and power in men but the results were statistically insignificant (123). These results suggest that the evening is the optimal timing to promote training-induced muscle hypertrophy. However, its mechanism remains unclear. In a recent animal study, it was reported that the preventive effects of stimulus like a rehabilitation on muscle atrophy depended on its timing (124). Intermittent weight-bearing for 4 h prevents the hindlimb-unloading-induced muscle atrophy and the up-regulation of *Atrogin1* expression, which is one of the muscle catabolic genes (124, 125). These preventive effects are greater in mice, which perform weight-bearing in the early active phase compared with weight-bearing in the late period of active phase (equivalent to evening in human because mice are nocturnal) (124). In addition, these preventive effects of weight-bearing at the early active phase are not observed in the *Clock* mutant mice, suggesting that the effects of rehabilitation time is mediated via the circadian clock protein CLOCK (124). From these reports, it is possible that the beneficial timing of exercise is different by your aim. Thus, exercise in the evening is better for the induction of muscle hypertrophy, while in the morning is better for the prevention of muscle loss. However, because there are few studies in this field called chrono-exercise, further studies are expected to generate strong and conclusive evidence.

### Lipid Metabolism

Endurance exercise controls the energy metabolism and oxygen (O_2_) consumption (126–128). Some studies show time-dependent or -independent response to acute endurance exercise (see below). In women, higher O_2_ consumption is observed during submaximal treadmill exercise in the afternoon and evening compared with morning (129). In the normal weight and obese men, fat oxidation during the incremental running exercise test is higher in the evening than in the morning, suggesting that evening exercise is better for fat burning (130). The beneficial effects of evening exercise on lipid metabolism in men are also observed in the change of post-exercise hormone levels (131). Treadmill running in the evening increases the free fatty acid levels, subsequent to the elevation of blood adrenaline and interleukin-6 levels, compared with morning running (131). In this report, exercise was performed under the postprandial condition in each time. On the other hand, in the other study, the exercise-induced fat oxidation for 24 h in men is only observed in cases where the exercise performed in the early morning before breakfast but not in the cases where exercise was performed after breakfast, in the afternoon, and evening. As one of its mechanism, it is suggested that it is easy to shift the fuel source from carbohydrate to fat because prior to breakfast, because it is the longer fasting condition within a day, resulting in depletion of energy derived from a carbohydrate source like glycogen (132). Similar responses are observed in women (133). Thus, acute response of fat oxidation to exercise is greater in the evening while the longer effects of exercise on fat oxidation are observed before breakfast.

## Summary and Perspectives

In this review, we confirm that the postprandial response of macronutrients is different based on the feeding time, when an identical meal is ingested at each time point. Circadian clock system and behavioral pattern are involved in the time-dependent physiological responses to each meal. Additionally, the metabolic function of macronutrients could be exacerbated by a misalignment between endogenous circadian clock and life cycle, as observed in shift workers (48, 134). On the other hand, there are a few reports to elucidate a chronic physiological effect of the distribution of macronutrients to each meal. Although there are many reports about the training time-dependent regulation of the day-night variation of athletic performance, the mechanisms are not fully understood and the research in these fields is only just beginning. Further evidence is expected to lead to a clearer understanding of the molecular mechanisms leading to the interaction between circadian clock and time-of-day effects. Finally, exercise also has the potential for a timekeeper of circadian clock, especially exercise at night-time induces phase-delay in humans (14). Thus, further evidence is needed to discuss the effects of exercise timing in the context of therapeutic effects and its circadian rhythms.

## Author Contributions

SA was involved in conceptualizing and writing the manuscript. SS was involved in conceptualizing and editing the manuscript.

### Conflict of Interest

The authors declare that the research was conducted in the absence of any commercial or financial relationships that could be construed as a potential conflict of interest.
